# Randomized clinical study on the analgesic effect of local infiltration versus spinal block for hemorrhoidectomy: Estudo clínico randomizado do efeito analgésico da infiltração local *versus* bloqueio espinal para hemorroidectomia

**DOI:** 10.1590/1516-3180.2017.0001260117

**Published:** 2017-05-29

**Authors:** Luis Antônio Borges, Plínio da Cunha Leal, Ed Carlos Rey Moura, Rioko Kimiko Sakata

**Affiliations:** I MD. Anesthesiologist, Hospital Municipal Dr. Mário Gatti, Campinas (SP), Brazil.; II MD, PhD. Professor, Department of Medical Practice, Universidade Federal do Maranhão (UFMA), São Luiz (MA), Brazil.; III MD, MSc. Professor, Department of Medical Practice, Universidade Federal do Maranhão (UFMA), São Luiz (MA), Brazil.; IV MD, PhD. Professor, Department of Anesthesia, Universidade Federal de São Paulo (Unifesp), São Paulo (SP), Brazil.

**Keywords:** Anesthesia, local, Anesthesia, spinal, Analgesia, Postoperative complications, Hemorrhoidectomy, Randomized controlled trial

## Abstract

**BACKGROUND AND OBJECTIVES:**

Postoperative analgesia and early recovery are important for hospital discharge. The primary objective of this study was to compare the analgesic effectiveness of perianal infiltration and subarachnoid anesthesia for hemorrhoidectomy. The secondary objective was to compare time to discharge, adverse effects and complications.

**DESIGN AND SETTING:**

Randomized, prospective and comparative study at Dr. Mário Gatti Hospital.

**METHODS:**

Forty patients aged 18-60, in American Society of Anesthesiologists physical status category 1 or 2, were included. The local group (LG) received local infiltration (0.75% ropivacaine) under general anesthesia; the spinal group (SG) received subarachnoid block (2 ml of 0.5% bupivacaine). Analgesic supplementation consisted of fentanyl for LG and lidocaine for SG. Postoperative pain intensity, sphincter relaxation, lower-limb strength, time to discharge, analgesic dose over one week and adverse effects were assessed.

**RESULTS:**

Eleven LG patients (52.4%) required supplementation, but no SG patients. Pain intensity was higher for LG up to 120 min, but there were no differences at 150 or 180 min. There were no differences in the need for paracetamol or tramadol. Times to first analgesic supplementation and hospital discharge were longer for SG. The adverse effects were nausea, dizziness and urinary retention.

**CONCLUSIONS:**

Pain intensity was higher in LG than in SG over the first 2 h, but without differences after 150 and 180 min. Time to first supplementation was shorter in LG. There were no differences in doses of paracetamol and tramadol, or in adverse effects.

## RESUMO

**CONTEXTO E OBJETIVO:**

A analgesia pós-operatória e a recuperação precoce são relevantes para a alta hospitalar. O objetivo primário deste estudo foi comparar a eficácia analgésica da infiltração perianal e da anestesia subaracnóidea para hemorroidectomia. O objetivo secundário foi comparar o tempo para alta, efeitos adversos e complicações.

**TIPO DE ESTUDO E LOCAL:**

Estudo randomizado prospectivo e comparativo, no Hospital Dr. Mário Gatti.

**MÉTODOS:**

Foram incluídos 40 pacientes com idades 18-60 anos, na categoria 1 ou 2 de status físico da Sociedade Americana de Anestesiologistas. O grupo local (GL) recebeu infiltração local (ropivacaína a 0,75%) sob anestesia geral; o espinal (GS) recebeu bloqueio subaracnóideo (2 ml de bupivacaína a 0,5%). A suplementação analgésica foi com fentanil para GL e lidocaína para GS. Foram avaliados: intensidade da dor no pós-operatório, relaxamento do esfíncter, força dos membros inferiores, tempo de alta, dose de analgésico em uma semana e efeitos adversos.

**RESULTADOS:**

Onze (52,4%) pacientes em GL necessitaram de complementação, e nenhum em GS. A intensidade da dor foi maior para GL até 120 minutos, sem diferenças em 150 ou 180 minutos. Não houve diferenças na necessidade de paracetamol ou tramadol. O tempo para a primeira complementação analgésica e a alta hospitalar foram maiores para GS. Os efeitos adversos foram náuseas, tonturas e retenção urinária.

**CONCLUSÕES:**

A intensidade da dor foi maior na GL que na GS nas primeiras 2 horas, porém sem diferenças após 150 e 180 minutos. O tempo para a primeira suplementação foi menor na GL; sem diferenças nas doses de paracetamol e tramadol e efeitos adversos.

## INTRODUCTION

Hemorrhoidectomy is often performed in outpatient settings. This surgical procedure can be conducted by means of local infiltration,^1^^,^^2^^,^^3^^,^^4^^,^^5^ in association with sedation and/or general anesthesia with pudendal nerve block,^4^^,^^6^^,^^7^ spinal block^5^^,^^6^^,^^8^ or epidural block,^9^^,^^10^ or with general anesthesia alone.^7^ The choice of anesthesia depends on the characteristics of both the disease and the patient, as well as professional experience.

Quick recovery, along with adequate and safe postoperative analgesia, is an important factor in relation to hospital discharge after any surgical procedure. The adverse effects and complications associated with the various techniques might increase the length of stay at the hospital, patient morbidity and healthcare costs.

Spinal anesthesia is widely used because of its simplicity, the quality of the analgesia obtained and the induction of anal sphincter relaxation that it provides, which is required for hemorrhoidectomy.^11^ However, this procedure is also associated with complications such as urine retention,^12^ with consequent discharge delay.

Perianal infiltration is a simple, easy-to-perform technique that is safer than spinal anesthesia because it does not involve the neuraxis. Long-acting local anesthetics are used to achieve longer analgesic effects. Some studies have shown that local infiltration with ropivacaine was effective for hemorrhoidectomy.^3^ However, other authors have used drug volumes that were too large (i.e. 40 ml for a 0.75% solution).^13^^,^^14^

No consensus yet exists regarding the efficacy of local infiltration, the duration of its effect or the associated adverse effects and complications. Therefore, studies that assess both the analgesic and adverse effects are necessary.

The primary objective of this study was to compare the analgesic effectiveness of perianal infiltration and subarachnoid anesthesia for hemorrhoidectomy. The secondary objective was to compare the time to discharge, the adverse effects and the complications.

## METHODS

### Study type and setting

This was a randomized controlled trial. Data were collected at Hospital Dr. Mário Gatti between December 2014 and November 2015.

### Ethics

This study was firstly approval by the ethics committee of Universidade Federal de São Paulo (CAAE 3714054.9000.5505). Patients undergoing hemorrhoidectomy were included in the study after they had signed an informed consent form. The study was registered at ClinicalTrials.gov (NCT02839538).

### Sample size

The sample size calculation was performed using SPSS for Windows. It was assumed that the response rate to the treatment tested would be a 30% reduction in pain intensity, with 95% power (beta), P = 0.05 (alpha) and an estimated standard deviation of 2.44. Therefore, the sample size would need to be 18 participants per group.^15^

### Participants

All patients undergoing hemorrhoidectomy in the same institution between December 2014 and November 2015 were included. The following patients were excluded from the study: those with associated conditions (fistula and fissure), infection of the puncture site, cognitive disorders, psychiatric illnesses, myocardial ischemia, arrhythmia or any other painful syndrome; those using anticoagulants or analgesics (within the last two weeks before the intervention); illicit drug users; and pregnant women.

### Randomization

Randomization was performed by an author who did not participate in the anesthesia and assessment, using the Randomizer software. The group assignment of each participant was placed inside an envelope numbered from 1 to 40. Participant allocation was performed via a draw, in which the envelopes were opened before the start of the intervention, at the surgical center. The participants were thus randomly selected to receive one type of anesthesia. It was impossible to read what was inside the envelopes. One surgeon performed all of the infiltration procedures, and one anesthetist performed both the general anesthesia for the local infiltration group (LG) and the spinal anesthesia for the spinal anesthesia group (SG). Another investigator who was not involved in the study evaluated the participants.

### Interventions

The participants were allocated to one of two groups. LG received surgery with local infiltration and general anesthesia, and SG received spinal block.

Monitoring during anesthesia was performed via pulse oximetry, cardioscopy, non-invasive blood pressure measurement and (in LG) capnography.

In LG, general anesthesia was administered using propofol (3 mg/kg), atracurium (0.5 mg/kg), propofol infusion (100 µg/kg/min), oxygen and a laryngeal mask. Next, the same surgeon performed local infiltration with 20 ml of 0.75% ropivacaine, which was injected between the internal and external anal sphincters using a 0.8 x 30-mm needle.

In SG, puncture was performed with the patients in a sitting position, using a 27G Quincke needle between L4 and L5 or between L5 and S1, with injection of 2 ml of 0.5% hyperbaric bupivacaine. After 10 min, the anesthesia was tested via the pinprick method, and patients whose score was zero on the pain scale proceeded to surgery.

Analgesic supplementation was performed as needed, using 50 µg of intravenous fentanyl in LG and infiltration of 5 ml of 1% lidocaine in SG. 

Postoperative analgesic rescue was initially performed using acetaminophen 500 mg/dose (maximum: 4 g/day). The cases without adequate relief 1 h later were given a tramadol dose of 50 mg.

### Outcomes

Upon discharge, the participants received a form, to be returned one week later, to record the following data: time of pain onset, amount of analgesics taken and adverse effects. Pain intensity was assessed on a numerical scale (from zero to 10).

The following outcomes were also assessed: need for intraoperative analgesic supplementation; sphincter relaxation (under conditions appropriate for surgery); postoperative pain intensity on a rating scale ranging from 0 to 10 at the end of surgery (T0) and every 30 min afterwards up to 180 min; dose of postoperative analgesic; time to first supplementation (from anesthesia to first dose); motor function of the lower limbs every 30 min until discharge in accordance with the Bromage scale (where 0 = no motor block; 1 = able to flex knees and move the feet but not lift the legs; 2 = able to move the feet only; and 3 = unable to move knees or feet); and time to hospital discharge (score 9-10 on Chung’s scale).^16^ The primary outcome was postoperative pain, and the secondary outcomes were adverse effects and time to discharge. Adverse effects and complications were also recorded.

### Statistical analyses

Statistical analyses were performed using SPSS for Windows. The following tests were applied: Mann-Whitney test for body mass index, pain intensity and first need for analgesic; chi-square test for gender, physical status and adverse effects; and Student’s t test for age, body weight, height, acetaminophen and tramadol dose, duration of surgery and time to discharge. The significance level was set at P ≤ 0.05.

## RESULTS

**Figure 1.**
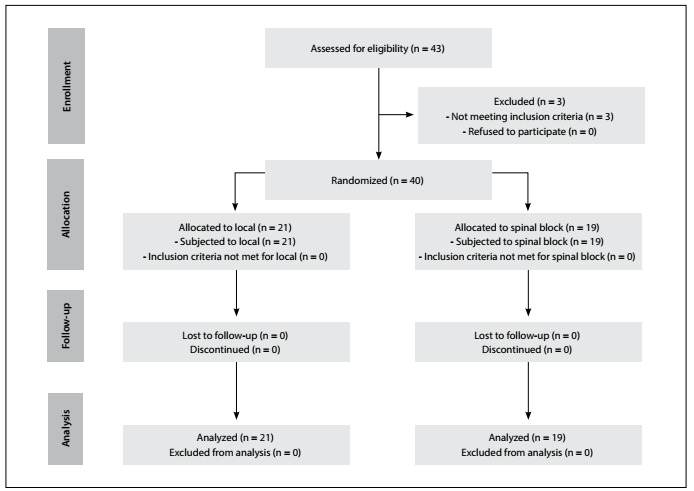
Consort flow diagram.

Forty patients of both genders, aged 18 to 60 years, who presented physical status 1 or 2 of the American Society of Anesthesiologists classification and were scheduled to undergo hemorrhoidectomy, were included in this study. The protocol sequence is shown in a flowchart (Figure 1). The groups did not differ significantly, with regard to their demographic data (Table 1) or duration of surgery (LG: 48.4 ± 2.9 min; SG: 57.8 ± 4.2; P = 0.07; Student’s t test). Sphincter relaxation was satisfactory for all participants.

**Table 1.**
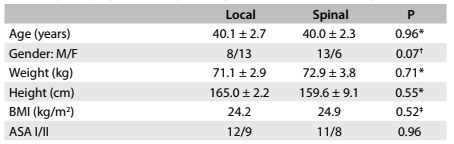
Sociodemographic and clinical characteristics of participants, according to age, gender, weight, body mass index and ASA physical status

BMI = body mass index; ASA = American Society of Anesthesiologists classification; ^*^Student’s t test (mean ± standard deviation); ^†^chi-square test; ^‡^Mann-Whitney test.

Eleven participants (52.4%) in LG (local group) (35.7 ± 42.3 µg) but none of the patients in SG required intraoperative analgesic supplementation with fentanyl.

The pain intensity was higher for LG at 0, 30, 60, 90 and 120 min after surgery, but there was no significant difference at 150 or 180 min (Table 2). Time to first analgesic supplementation was longer for SG (Table 3). The groups did not differ with regard to their use of acetaminophen or tramadol during the first week after surgery (Table 3).)

**Table 2.**
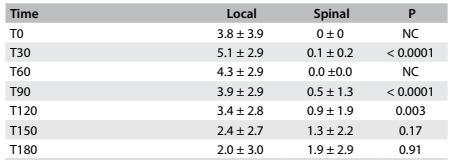
Intensity of pain at each 30 minutes, according to numerical scale (mean ± standard deviation)

Student’s t test; NC = not calculated; T0 = end of surgery.

**Table 3.**
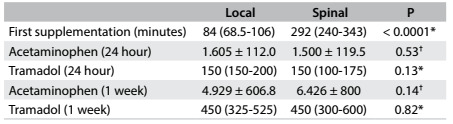
Time that elapsed until first postoperative supplementation (after infiltration or spinal block), expressed as mean (minimum-maximum); doses of acetaminophen (mean ± standard deviation, SD); and doses of tramadol (mg) expressed as mean (minimum-maximum)

^*^Mann-Whitney test, expressed as mean (minimum-maximum); ^†^Student’s t test (mean ± SD).

All of the participants in SG scored zero on the Bromage scale, 210 min after the end of surgery. The time to discharge, calculated from the onset of anesthesia, was longer for SG (LG: 241.9 ± 8.1 min; SG: 347.5 ± 27.6 min; P = 0.0005; Student’s t test).

The participants reported the following adverse effects: nausea (LG: 4; SG = 0; P = 0.14; chi-square test), dizziness (LG: 1; SG: 0; p = 0.340; chi-square test) and urine retention (LG: 0; SG: 4; P = 0.09; chi-square test).

## DISCUSSION

The pain intensity was higher for LG than SG over the first 2 h after hemorrhoidectomy. The time to the first analgesic supplementation was significantly shorter, with no difference in the analgesic supplementation or in adverse effects.

This study investigated infiltration because this technique is simple, and recovery is quick; thus, it is appropriate for surgery in outpatient settings. Infiltration alone might promote an adequate level of analgesia for surgery, but patients remain able to perceive the surgical manipulation. This feeling is often uncomfortable; therefore, medication needs to be administered, to sedate the patients. Infiltration can be performed in combination with sedation,^17^ or with general anesthesia as in this study. Another study has also combined these methods.^18^ Like us, the authors of a previous study^8^ used a laryngeal mask to combine general anesthesia with fentanyl, propofol and spinal anesthesia, in order to promote greater patient comfort during local infiltration of anesthetic. In the present study, only fentanyl was used for supplementation, because this was sufficient to maintain postoperative analgesia.

According to the authors of one study, the quality of postoperative analgesia is better when the full posterior perineum is blocked. However, the technique involved is more complex, and higher doses of anesthetics are used.^14^ Local anesthetic can be absorbed, causing toxic effects that make it impossible for clinicians to administer large doses, particularly regarding bupivacaine.

As in other studies,^13^^,^^14^ we administered long-acting 0.75% ropivacaine, which is less toxic than bupivacaine. On the other hand, ropivacaine causes vasoconstriction, which limits its blood absorption.^19^ Alternatively, short-acting lidocaine^17^ or bupivacaine^12^ can also be used.

The volume of anesthetic reported in the literature has varied widely, from 6 ml^9^ to 20 ml^10^ and 40 ml.^13^^,^^14^ We used an intermediate dose of 20 ml.

The peak plasma concentration is achieved 15 min after ropivacaine infiltration into the subcutaneous tissue.^20^^,^^21^ However, no previous study has reported local infiltration for treating hemorrhoidectomy.

In another study, 47% of the patients reported pain and discomfort during surgery. However, the dose used was small (6 ml of 0.25% bupivacaine),^22^ compared with what was used in the present study.

Local infiltration with 0.25% bupivacaine has been reported to promote excellent sphincter relaxation.^22^ In the present study, the sphincter relaxation obtained in all of the participants was adequate for surgery.

In one study, there was no difference in pain score after 24 h, after local or spinal anesthesia, except at the 6-h assessment, when the intensity was higher for the group that received spinal anesthesia. Postoperative analgesia was excellent in more than 90% of the participants who received local infiltration, but was excellent in less than 50% of the group that received a spinal block.^5^ Another study did not find any differences in pain intensity between the local infiltration and spinal anesthesia groups at 6 and 24 h after surgery; however, the latter group required more analgesic rescue treatment.^12^

In one study in which bupivacaine infiltration was performed in combination with general anesthesia, the analgesic effect lasted for approximately 10 h,^18^ i.e. much longer than the effect in the present study (i.e. 84 min).

It should be noted that this study presents the limitation that it was not possible to blind the groups.

In this study, the length of hospital stay was shorter for LG than for SG, which corroborates the results reported in the literature.^5^ Longer stays after spinal anesthesia for hemorrhoidectomy have been correlated with urine retention, pain and bleeding.^23^

In this study, urinary retention occurred in 19% of SG. However, the incidence of this complication reported in the literature is higher: between 30%^12^ and 36%.^5^ Motor block of the lower limbs might prolong the hospital stay.

Following spinal anesthesia, the reported rate of headaches is 24%.^5^ In the present study, nausea was reported by 19% of LG, whereas the analgesic rescue dose did not differ between the groups. One study that used perianal infiltration did not detect any complications.^24^ Intraoperative arterial hypotension occurred in another study,^12^ but did not occur in the present study.

## CONCLUSION

In this study, local infiltration showed less postoperative analgesic efficacy, but recovery was faster. In clinical practice, analgesia might be enhanced through preventive multimodal combination of analgesics at the end of surgery. Infiltration might be an alternative option for patients undergoing hemorrhoidectomy.
